# Antibiotic Use among Patients Visiting Primary Hospitals in Northwest Ethiopia: A Multicenter Cross-Sectional Survey

**DOI:** 10.1155/2022/2306637

**Published:** 2022-09-10

**Authors:** Adeladlew Kassie Netere, Ashenafi Kibret Sendekie

**Affiliations:** Department of Clinical Pharmacy, School of Pharmacy, College of Medicine and Health Sciences, University of Gondar, Gondar, Ethiopia

## Abstract

**Background:**

Antimicrobial medications are becoming ineffective because of the surge in antimicrobial resistance. Poor knowledge and inappropriate beliefs combined with the misuse of antibiotics may be common in the community and public health institutions. This study assessed the knowledge, belief, and antibiotic use practice among patients visiting rural hospitals in Northwest Ethiopia.

**Methods:**

A facility-based multicenter cross-sectional survey was conducted in Northwest Ethiopian primary hospitals from August to September 2020. The data are presented as frequencies and means (±SD) of our findings. The independent samples *T* test and One-Way Analysis of Variance (ANOVA) were used to explore the mean knowledge differences of antibiotic use among respondents. A *p*value of <0.05 with 95% CI was considered significant.

**Results:**

More than half of the participants (57.2%) were males, and the mean age was 34.8 ± 13.4 years. The mean (±SD) knowledge score of the respondents was 3.69 (±1.7) (range: 0 to 10), and the majority (69.7%) had poor knowledge. The mean (±SD) belief score (acceptance towards antibiotic use) of the respondents was 20.08 ± 4 (range: 7 to 35) and most (70.1%) of the respondents had moderate levels of perception regarding antibiotic use. The majority (69.5%) of respondents took antibiotics over the past year. Participants practiced inappropriate uses of antibiotics such as medication discontinuation (49.5%), keeping unused antibiotics for future use (35.5%), and sharing medications with/from others (30.1%). Diploma holder participants had significantly higher mean knowledge scores on antibiotic use than those who were unable to read and write (*p*=0.047). Similarly, students had higher mean knowledge scores compared with farmers (*p*=0.024), merchants (*p*=0.031), and housewives (*p*=0.047).

**Conclusion:**

Our study showed a widespread misuse of antibiotics in Northwest Ethiopia. Malpractice such as dose interrupting, sharing of antibiotics for/from the others, and keeping leftover drugs for future use were common among the respondents. These findings suggest that a greater effort is required in public education related to proper and safe uses of antibiotics and that greater efforts are needed to enforce the control of antibiotic use to overcome the emergence of antibiotic resistance.

## 1. Introduction

The global increase in antimicrobial resistance (AMR), which frequently leads to unforeseen life-threatening complications [1, 2], is associated with the misunderstanding and malpractice of antibiotic use [3, 4]. Various reasons, including inadequate knowledge and misuse of antibiotics, contribute to increases in AMR [5, 6]. The global AMR “superbug crisis” requires a multidimensional worldwide approach [1, 7], with improved public knowledge and behaviors [2].

Studies in both industrialized and nonindustrialized countries reported lack of basic knowledge on appropriate antibiotics use[8–10]. A study from Ethiopia revealed that the community knowledge and attitude towards antibiotic use were poor, and the nonprescription use of antibiotics was also common [11]. The existing misconceptions on the effectiveness and indications of antibiotic use suggested that public perceptions should be considered when efforts are made to better inform the public and prescribers on the safe and effective use of antibiotics [12]. An important consideration is the patients' expectation of prescriptions when visiting physicians or medical clinics [13]. The previous study disclosed that the patients who used antibiotics without guidance made inappropriate choices about the antibiotics due to insufficient knowledge [7]. Besides, many patients believe that antibiotics can improve outcomes from both bacterial and viral infections [14, 15].

Several studies on antibiotic misuse recommended issues related to poor patient knowledge, attitudes and practices on the appropriate use of antibiotics [16, 17], with suggestions towards adhering improved guidelines on rational antibiotic use practices [18, 19]. This tradition may assist both patients and prescribers with customized innovations given that safe and effective use of antibiotics has improved [20]. The irrational use of antibiotics by both patients and healthcare providers is common in developing nations such as Ethiopia, where medication sharing and nonadherence are collective challenges. According to the Food, Medicine, and Healthcare Administration and Control Authority of Ethiopia (FMHCA), 70% of patients who visited outpatient clinics had ∼ 40% of irrational prescriptions for antibiotics [21]. Data on the rational uses of antibiotics and common practices are more accessible [9, 10]. However, such information is scarce for developing nations like Ethiopia. This study assessed the knowledge and use of antibiotics by patients visiting primary hospitals in rural districts of Northwest Ethiopia.

## 2. Methods

### 2.1. Study Design and Setting

A facility-based multicenter cross-sectional survey was conducted from August to September 2020 in primary hospitals in Northwest Ethiopia. The study area included five randomly selected public primary hospitals called Addis Zemen, Chilga, Debark, Kolladiba, and Wogera in the three Gondar zones, where there were ten primary hospitals. Based on the hospital's annual records, there were about 163,234 patients served by the year 2020 in their outpatient departments, given that Addis Zemen, Chilga, Debark, Kolladiba, and Wogera were delivering services to 41,208, 27,472, 40,804, 26,260, and 27,472 clients, respectively [22]. This institutional-based study involved outpatients who were able to be interviewed. All volunteer clients aged 18 and above and patients served in the outpatient department were included in the study, whilst those experiencing difficulties being interviewed with the questionnaire-based approaches or who were unable to provide consent because of serious illness, cognitive impairments, and communication problems were excluded.

### 2.2. Sample Size Determination

**Figure 1:**
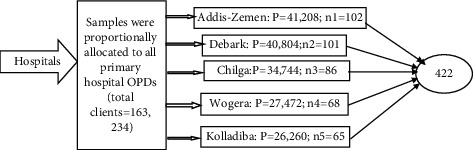
Schematic representation diagrams of the allocation of respondents per institution (*P* = number of patients visiting hospital site; *n* = patients enrolled in the study).

A sample size of 422 individuals from the outpatient department (OPD) service users was included by assuming 50% of the maximum correct responses to questions on knowledge, belief, and practices of antibiotic use. A 5% absolute precision or margin of error, 5% significance, and 95% confidence level were used; and 10% of contingency for the nonresponses was applied. A multi-stage sampling strategy was employed in which the study participants were allocated proportionally from each randomly selected hospital. Finally, the study subjects were nominated through a systematic random sampling method, where 102, 86, 101, 65, and 68 respondents were involved in Addis Zemen, Chilga, Debark, Kolladiba, and Wogera, respectively (Figure 1). Respondents were allocated proportionally as per the number of patients served in the outpatient departments in all hospitals, which were initially selected by a simple random sampling method.

### 2.3. Data Collection Instruments and Procedures

The English version of the questionnaire was translated in to Amharic, which is the official local language, and which was then translated back to English again to maintain consistency. A pretest was done on 5% of clients having similar characteristics in one of the selected hospitals and excluded from the final analysis. Further, local specialists were involved in the evaluation of item validity. The questionnaire contains items measuring the clients' knowledge, beliefs, and practices related to antibiotic use and were structured based on published articles with some modifications [22–25]. The questionnaire then underwent minor adjustments based on the pretest results prior to actual data collection. Two data collectors were trained for the objectives of the study and familiarized with the data collection tool and approaches. Sequential steps of participant enrollment and consent procedures were provided to the data collectors and used during the data collection procedures. The interview was conducted in the respondents' official language and lasted an average of 20 to 25 minutes. The interview-based questionnaires included sociodemographic characteristics, knowledge of antibiotic uses, allergies and resistance, self-reported practices of antibiotics use, levels of satisfaction with the medical services provided by health professionals, ability to differentiate different antibiotic and the sources of information regarding antibiotic use.

Ten items assessed the knowledge of participants on antibiotics using “Yes”, “No”, and “Don't know” responses. Seven questions addressed the beliefs of the respondents. A five-point Likert scale was used (where 1, 2, 3, 4, and 5 represented strongly disagree, disagree, uncertain, agree, and strongly agree, respectively) to measure responses to belief questions. Items regarding practices of antibiotics use were started by dichotomized questions to assess experiences with antibiotic use, i.e., “Have you taken any antibiotics in the last one year? (Yes/No)”. The “Yes” response was then verified by asking open- and closed-ended questions. The questions used in the study are summarized in Table 1.

We assigned “No” as the correct response for Q1-7 and “Yes” as right response for Q8-10. Those who scored below 50% in responses to these ten questions had poor knowledge, and those scored 50%–75% had moderate knowledge and those who scored more than 75% had adequate knowledge [26]. For “Belief” items, we considered “disagree” as the correct response. Scores of 1, 2, 3, 4, and 5 were assigned for strongly disagree, disagree, uncertain, agree, and strongly agree, respectively. A total score ranged from 7 to 35 for the seven “Belief” questions. Unlike knowledge scores, respondents scoring below 50% were designated as appropriately believed, 50–75% as moderately believed, and those scoring above 75% as inappropriately believed [26].

### 2.4. Statistical Analysis

The data were entered and analyzed using IBM SPSS Statistics for Windows, version 22.0. Sociodemographic characteristics with categorical variables were described by frequency and percentage. The mean and standard deviation (SD) were used for continuous variables. Independent-samples *T*-test and one-way ANOVA were employed to explore mean knowledge differences in antibiotic use among respondents. A *p*-value of <0.05 with a 95% CI was considered significant.

### 2.5. Ethical Approval and Consent to Participate

This study was approved by the School of Pharmacy Research Ethics Review Committee, University of Gondar (Approval Number: UoG-SOP157/2018). We also obtained both verbal and written informed consent from all the respondents before the start of each interview after explaining the purpose of the study. The information obtained from the study was not disclosed to any third party, and code numbers were used to identify study participants.

## 3. Results

### 3.1. Socio-demographic Characteristics of the Participants

A total of 422 subjects completed and returned the survey questionnaire; with the majority (57.2%) being males aged 34.8 ± 13.4 years. Farmers represented about 33% of the study participants. More than half visited the health facilities either once (25.4%) or twice (26.5%) within a year. Sixty percent of those surveyed rated the healthcare services provided by health professionals as satisfactory. The largest (24.2%) and smallest (15.4%) groups of participants were recruited from Addis-Zemen and Kolladiba primary hospitals, respectively (Table 2).

### 3.2. Knowledge of Antibiotic Use

**Figure 2:**
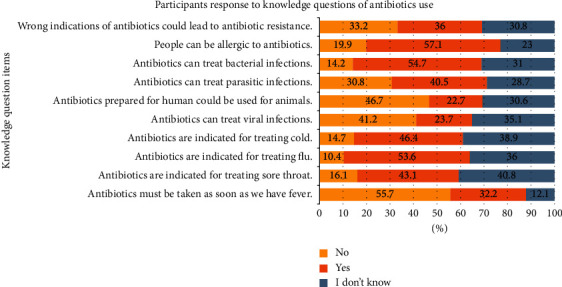
Study participants' antibiotic use knowledge levels for each question item.

More than half (57.1%) of the respondents reported that they might be allergic to antibiotics (Figure 2). Moreover, most study participants indicated bacterial infections could be treated by antibiotics (54.7%), while more than half (55.7%) responded incorrectly that antibiotics must be taken as soon as a fever develops. Further, nearly half (46.7%) of the respondents wrongly reported that antibiotics for the treatment of humans could also be used in animals. The mean (±SD) knowledge score was 3.69 (±1.7) (range: 0 to 10). The question items related to knowledge of antibiotic use indicated that 69.7% of study participants had poor levels of knowledge, 28.7% had moderate levels of knowledge, and only 1.7% had adequate levels of knowledge.

### 3.3. Beliefs of Antibiotic Use

**Figure 3:**
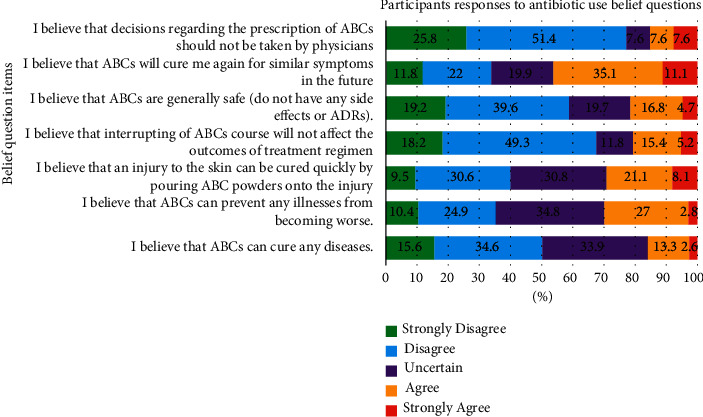
Participant belief on the antibiotic use.

The overall mean (±SD) belief score of the respondents was 20.08 (±4), with a range of 7 to 35 (potential ranges from 7 to 30), of which 23.9% held appropriate beliefs. The majority of the study group (70.1%) held moderate beliefs, while 5.9% held inappropriate beliefs about the use of antibiotics. Slightly more than half (51.4%) of the study subjects held appropriate beliefs that the prescription of antibiotics must be provided by physicians, whereas more than one-third (35.1%) held inappropriate beliefs given that antibiotics will prevent similar symptoms in the future. On the other hand, more than one-third (34.8%) of the surveyed subjects were uncertain about whether antibiotics could prevent any illnesses from becoming worse or if an injury could be cured more quickly by pouring antibiotic powder onto the injury site (Figure 3).

### 3.4. Antibiotic Use Practices

More than two-thirds (64.7%) of those interviewed commented that they had taken antibiotics in the past year. Among these, amoxicillin (36.3%) was the single most used medication, followed by ampicillin and metronidazole (8.8% for each medication). Cough (19.4%), wounds (10.7%), and diarrhea diseases (10.2%) were the primary reasons for using antibiotics. Nearly, a third (30.1%) of respondents shared medications for/from somebody else. Almost half (49.5%) of the study participants discontinued antibiotic medications once their symptoms subsided, while more than a third (35.5%) of participants kept unused antibiotics for future use. Moreover, about eleven percent of the participants replied that they took antibiotics without a prescription (Table 3).

### 3.5. Antibiotics Awareness

Amoxicillin and ampicillin were the most known antibiotics to 36.7% and 14% of participants, respectively. Despite almost one-fourth (24.9%) of the surveyed being capable enough to differentiate between four and above antibiotics displayed, a significant number (59.5%) were unaware of their importance. Most (83.6%) of the participants responded that appropriate antibiotic use should be determined by physicians (Table 4).

### 3.6. Knowledge Difference among Participants on Antibiotic Uses

A one-way ANOVA test indicated that there were significant differences in the mean knowledge scores of participants regarding their educational levels they held (*F* = 2.3, *p*=0.043) and occupational types (*F* = 3.3, *p*=0.011). The Tukey posthoc test also revealed that diploma-graduated participants had significantly higher knowledge scores (mean = 3.95) than those who were unable to read and write (mean = 3.3) (*p*=0.047) about antibiotic use. Concerning the occupational types of study participants, students had significantly higher mean knowledge scores (mean = 4.3; *p*=0.024) than farmers (mean = 3.5; *p*=0.031), merchants (mean = 3.3; *p*=0.05), and housewives (mean = 3.5; *p*=0.047) (Table 5).

## 4. Discussion

The principles of appropriate antibiotic use practices have been encouraged, with adherence being more important than ever. The disheartening fact is that antibiotics are often used incorrectly. The downturn of new antibiotic development combined with inappropriate use poses unanticipated challenges to the availability of effective therapies [27]. The use of antibiotics in low- and middle-income countries such as Ethiopia has increased during the last twenty years [28, 29]. We investigated the knowledge, beliefs, and practices regarding the antibiotic use of patients in resource-limited settings. Our findings suggest that most study participants had poor levels of knowledge and moderate levels of belief, combined with widespread misuse of antibiotics.

The findings of this study demonstrated that just fewer than three-quarters of study participants had poor levels of knowledge of antibiotic use, with an overall mean score of 3.7 out of 10 points. The knowledge levels in this study are similar to previous findings [11, 30–32]. This insufficient knowledge of antibiotic uses in the low- and middle-income countries (LMICs) might be related to limited resources on antibiotic prescriptions and lower levels of professional and authority monitoring. Moreover, in relation to antibiotic resistance, just under two-thirds of the study participants were unaware of antibiotic resistance, as also reported by others [33, 34]. This poor awareness of the appropriate use of antibiotics has been identified as a major cause of antibiotic resistance [35–37]. This finding is in contrast to an earlier study conducted in Hara city, in the eastern part of Ethiopia, where the participants had better information on antibiotics resistance [11]. This inconsistency might be related to differences in study settings and is likely because the residents of Harar city were better informed about the consequences of antibiotic misuse. Significant numbers of the study participants (40–56%) believed that antibiotics could be taken regardless of fever type and could be used to treat flu, sore throats, and parasitic infections. This is in good agreement with the previous studies [11, 33, 38]. The results of this study suggest that patients dwelling in rural districts could be with insufficient knowledge regarding the appropriate use of antibiotics. The likelihood reason might be the patients do lack resources and have limited public awareness of the appropriate use of antibiotics. Our study also suggests that hospital healthcare providers, the supporting zonal health bureaus, and others could provide improved health education and resources, and promote the appropriate use of antibiotics by people living in rural districts of resource-limited settings.

Our study also indicates that significant numbers of the study participants had moderate levels of belief in antibiotic use. Almost more than two-thirds of study participants believed that interruption of the antibiotic treatment could affect the therapeutic outcomes, and most participants agreed and/or strongly agreed that the prescription of antibiotics must be taken by physicians. These findings are in complete agreement with a previous report from Ethiopia [11] which concluded that interrupting antibiotic use probably affected treatment outcomes and led to antibiotic resistance. Nearly, half of the participants in our study also believed that antibiotics could cure them again for similar medical symptoms in the future, likely because of their inadequate knowledge of antibiotic use. Nonetheless, these results differ from an earlier report from Harar city [11], in part because of differences in study populations and their access to information on antibiotic use.

Our study indicates that significant numbers of the study participants had used antibiotics in the past year and then shared them with others, which concurs well with earlier studies [7, 11]. Amoxicillin was the most frequently used medication (36.7%), followed by ampicillin (14%) and metronidazole. The findings of our study also indicated widespread malpractice of antibiotic consumption that was attributed to using antibiotics without a medical prescription, sharing medications, and discontinuing and keeping unused antibiotics for future use. This lends support to previous results [39, 40]. These findings could be due to poor levels of knowledge, the inappropriate beliefs about antibiotic use, and consumption trends [41]. In addition, the poor healthcare systems in developing countries could also be an important contributor to these malpractices [42].

Study participants who held a diploma educational level had higher mean knowledge scores on antibiotic use than those who were unable to read and write. A study from Bahir Dar (Ethiopia) also indicated that lower education status was associated with lower knowledge levels and contributed to the inappropriate use of antibiotics [41]. Likewise, students had higher knowledge scores than farmers, merchants, and housewives, suggesting that students could be trained to inform their communities on the safe and effective use of antibiotics.

### 4.1. Study Limitations

This study has some limitations, including: (1) the results of the study may not be generalized for all populations in Ethiopia because the study was carried out only in rural districts; (2) the data collection was based on the recall of the study participants; and (3) there were no laboratory data on the details of antibiotic resistance (e.g., multidrug resistance).

## 5. Conclusion

This study describes the widespread malpractice of antibiotic use in rural Ethiopia, where study participants had limited knowledge of the appropriate use of antibiotics. We documented different types of misuse of antibiotics, such as consuming antibiotics without medical prescriptions, dose interruptions, sharing of antibiotics for/from others, and keeping leftover drugs for future use. Greater efforts in education and training could reduce the inappropriate use of antibiotics and potentially limit antibiotics. Enforcing antibiotic prescription regulations could also be considered to control the use of antibiotics without prescriptions.

## Figures and Tables

**Table 1:** Knowledge and belief items on antibiotic use in interview-based questions to respondents in Northwest Ethiopia primary hospitals.

*Items of knowledge questions*
Q1. Antibiotics must be taken as soon as we have fever.
Q2. Antibiotics are indicated for treating sore throat.
Q3. Antibiotics are indicated for treating flu.
Q4. Antibiotics are indicated for treating cold.
Q5. Antibiotics can treat viral infections.
Q6. Antibiotics prepared for human could be used for animals.
Q7. Antibiotics can treat parasitic infections.
Q8. Antibiotics can treat bacterial infections.
Q9. People can be allergic to antibiotics.
Q10. Wrong indications of antibiotics could lead to antibiotic resistance.

*Items of belief questions*
Q1. I believe that antibiotics can cure any diseases.
Q2. I believe that antibiotics can prevent any illnesses from becoming worse.
Q3. I believe an injury to the skin can be cured quickly by pouring antibiotic powders onto the injury.
Q4. I believe that interrupting the course of antibiotic use will not affect the outcomes of treatment regimen
Q5. I believe that antibiotics are generally safe (do not have any side effects or ADRs).
Q6. I believe that antibiotics will cure me again for similar symptoms in the future
Q7. I believe that decisions regarding prescription of antibiotics should not be taken by physicians

**Table 2:** Sociodemographic characteristics of participants at the primary hospitals of Northwest Ethiopia (*N* = 422).

Variables		Frequency (%)
*Sex*	Male	244 (57.2)
	Female	178 (42.8)

*Age in years*	Mean (±SD)	34.79 ± 13.41
*Location*	Urban	211 (50)
	Rural	211 (50)

*Marital status*	Single	134 (31.8)
	Married	248 (58.8)
	Ever married	40 (9.5)

*Educational status*	Unable to read and write	138 (32.7)
	Read and write only	55 (13)
	Primary education (1–8 grades)	56 (13.3)
	Secondary education (9–12 grades)	57 (13.5)
	Diploma and vocational training	75 (17.8)
	Degree and above/University	41 (9.7)

*Occupational status*	Farmer	136 (32.2)
	Merchant	38 (9)
	Employee	99 (23.5)
	Student	75 (17.8)
	Housewife	774 (17.5)

*Healthcare visits/year*	Not at all	28 (6.6)
	Once	107 (25.4)
	Twice	112 (26.5)
	Three time	59 (14)
	Four times	41 (10.7)
	Five or more times	71 (16.8)

*Satisfaction levels with health professionals*	Unsatisfied	93 (22)
	Indifferent	76 (18)
	Satisfied	253 (60)

*Hospital location*	Addis zemen	102 (24.2)
	Debark	101 (23.9)
	Chilga	86 (20.4)
	Wogera	68 (16.1)
	Kolladiba	65 (15.4)

**Table 3:** Participant responses to antibiotic use practice questions.

S. no	Practice questions		Frequency (%)
1	*Have you taken any antibiotics in the last year?*	Yes	273 (64.7)
		No	149 (35.3)

2	*Have you ever shared your medications with/from somebody else?*	Yes	127 (30.1)
		No	295 (69.9)

3	*Which antibiotics have you used? (n* *=* *291 (69%))*	Amoxicillin	153 (36.3)
		Ampicillin	37 (8.8)
		Metronidazole	37 (8.8)
		Ciprofloxacin	24 (5.7)
		Doxycycline	16 (3.8)
		Tetracycline	15 (3.6)
		Chloramphenicol	9 (2.1)

4	*Who used the drug (n* *=* *301)*	Myself	256 (60.7)
		Family members	45 (10.7)

5	*For what health problem did you use the drug(s)? (n* *=* *289)*	Cough	82 (19.4)
		Diarrhea	43 (10.2)
		Fever	36 (8.5)
		RTI	13 (3.1)
		Wound	45 (10.7)
		UTI	16 (3.8)
		Headache	27 (6.4)
		Colic pain	21 (5.0)
		Other	6 (1.4)

6	*Where did you get the drugs? (n* *=* *298)*	From health professional prescription	262 (62.1)
		Directly bought from pharmacy	30 (7.1)
		Lend from other family member, neighbor	1 (0.2)
		Bought from nonpharmacy source	5 (1.2)

7	*Did you discontinue therapy once your symptoms subsided? (n* *=* *301)*	Yes	209 (49.5)
		No	92 (21.8)

8	*Do you keep leftovers antibiotics for future use?*	Yes	150 (35.5)
		No	272 (64.5)

9	*How often do you use nonprescribed antibiotics?*	Never	238 (56.4)
		Rarely	138 (32.7)
		Often	33 (7.8)
		Very often	13 (3.1)

**Table 4:** Antibiotic awareness by participants at primary hospitals of Northwest Ethiopia.

S. no	Items		Frequency (%)
1	*Among the list, which drug(s) you know?*	Amoxicillin	155 (36.7)
		Ampicillin	59 (14.0)
		Augmentin	21 (5.0)
		Tetracycline	31 (7.3)
		Ciprofloxacin	34 (8.1)
		Cotrimoxazole	18 (4.3)
		Metronidazole	36 (8.5)
		Doxycycline	10 (2.4)
		Chloramphenicol	10 (2.4)
		None	48 (11.4)

2	*Can you mention its (their) importance?*	I do not know	251 (59.5)
		Other disease	19 (4.5)
		Infection	100 (23.7)
		Trauma	20 (4.7)
		Headache and pain	32 (7.6)

3	*Ability to differentiate displayed antibiotics*	Unable to differentiate	58 (13.7)
		Able to differentiate one	85 (20.1)
		Able to differentiate two	93 (22.0)
		Able to differentiate three	81 (19.2)
		Able to differentiate four and above	105 (24.9)

4	*Appropriate antibiotic use duration (period) should be indicated by*	Physician or pharmacist (1)	353 (83.6)
		Drug leaflet (2)	14 (3.3)
		Until disappearance of symptoms (3)	15 (3.6)
		Relief of symptoms (4)	18 (4.3)
		Family members or friends (5)	14 (3.3)
		One and two	5 (1.2)
		One, two, and three	3(0.7)

5	*Who is the source of your information?*	Relatives	19 (4.5)
		Friends	18 (4.3)
		Health professionals	377 (89.3)
		Mass media	8 (2.1)

**Table 5:** Mean knowledge score differences among the respondents regarding antibiotics use illustrated with the independent-samples *T* test and One-Way ANOVA analysis table.

Variables	Category	*Overall score of knowledge of antibiotic use*		
		Mean (±SD)	T/F	*P*value
*Sex*	Male	3.6 (1.6)	−0.74^*∗*^	0.460
	Female	3.8 (1.8)		

*Residence*	Urban	3.8 (1.6)	0.867^*∗*^	0.386
	Rural	3.6 (1.8)		

*Educational status*	Unable to read and write	3.3 (1.7)	2.3^*∗∗*^	**0.043**
	Read and write only	3.9 (1.7)		
	Primary education	3.8 (1.6)		
	Secondary education	3.96 (2.1)		
	Diploma	3.95 (1.5)		
	Degree and above	3.7 (1.2)		

*Occupation types*	Farmer	3.5 (1.6)	3.3^*∗∗*^	**0.011**
	Merchant	3.3 (1.6)		
	Employee	3.8 (1.5)		
	Student^a^	4.3 (1.8)		
	House wife	3.5 (1.9)		

*Marital status*	Single	3.9 (1.7)	2.4^*∗∗*^	0.096
	Married	3.5 (1.7)		
	Ever married	3.85 (1.5)		

*Belief of antibiotic use*	Strong belief	3.8 (1.8)	0.6^*∗∗*^	0.572
	Moderate belief	3.7 (1.7)		
	Weak belief	3.4 (1.6)		

^*∗*^ *T*-Independent-samples the *T*-test was used for variables with two categories; ^*∗∗*^F-One-Way ANOVA was used for variables with three or more categories; ^a-^students (individuals who were attending school at primary, secondary, and higher institutions); bold values denote significant differences (*p* < 0.05).

## Data Availability

All necessary data are available in the manuscript. Further data will be available from the corresponding author upon reasonable request.

## Abbreviations

AMR: Antimicrobial resistance.
